# Motor inhibition during voluntary gait initiation in young and older adults

**DOI:** 10.1038/s41598-024-79790-5

**Published:** 2024-11-15

**Authors:** Eunyoung Kwag, Igor Komnik, Dominic Bachmann, Wiebren Zijlstra

**Affiliations:** Institute of Movement and Sport Gerontology, German Sport University Cologne, Am Sportpark Müngersdorf 6, 50933 Cologne, Germany

**Keywords:** Mobility, Balance, Cognition, Executive functions, Stop-signal tasks, Ageing, Cognitive ageing, Motor control, Ageing

## Abstract

Based on a novel approach, this study explores feasibility and relevance of an inhibition task for studying age-related differences in motor inhibition during gait initiation. When presented with a go-signal, young adults (YA, n = 24) and older adults (OA, n = 55) were required to promptly initiate gait. Participants completed 3 blocks of 12 gait trials. Each block contained 3 stop trials in which the go-signal was followed by a stop-signal that required the person to block gait initiation and remain standing. Stop-signals were presented randomly and with different delays. Data analyses focused on changes in the centre of pressure (COP) and success of motor inhibition. Compared to go-trials, stop trials resulted in a marked decrease of timing and amplitude of COP displacement. Overall success rate of motor inhibition was low (29% in YA vs. 19% in OA) and decreased with increasing COP displacement. Inhibitory success was associated with two strategies: a pro-active cautious COP displacement; and the inhibition of further COP displacement after a stop-signal. Results demonstrate age-related differences in adaptive behavior as well as boundaries beyond which neither old nor young persons were successful. This study yields important insights into motor inhibition during gait and essential input nto further studies.

## Introduction

Mobility in daily life may present various unanticipated challenges such as potential collisions with obstacles, other pedestrians or traffic. Thus, safe progression requires one to continuously perceive the environment and adapt locomotion if needed. Such adaptive control is possible based on cognitive processes, so-called executive functions^1^, that enable a person to monitor his/her behavior in relation to the environment and adapt behavior in accordance with individual goals. Hence, executive functions are crucial for adaptive locomotion. One of the key components of executive functions is inhibitory control (IC), which among others comprises selective attention as well as the ability to modify or suppress actions in order to achieve goals^1–3^. Systematic reviews show that age-related changes in executive functioning are associated with reduced mobility and an increased fall-risk^4,5^. Additionally, recent studies highlight the importance of IC for gait adaptability, balance performance, and preventing falls^6–8^.

Though the above information indicates the relevance of executive functions for mobility, present knowledge is mostly based on correlative studies which relate the performance of standardized cognitive tests -which do not challenge balance- to the performance of balance or gait tasks and fall risk. For example, IC is often assessed using cognitive tasks that require verbal responses (as in the Stroop Color and Word test^9^) or key press responses (as in Go-No-Go, or Stop-signal tasks^2^). Our recent scoping review^7^ investigating balance tasks that integrate IC showed that only a few studies are available in older adults (OA) and that the majority of these few studies focused on perceptual inhibition (i.e. resolving conflicting stimuli followed by initiating a correct response (cf. cognitive inhibition^10^) rather than on motor inhibition. Since motor inhibition, i.e., suppressing an incorrect motor action (behavioral inhibition^10^), is essential for safe locomotion, we aimed to develop a gait-related task that allows to study age-related differences in the performance of a gait task that requires inhibitory control. Similar to usual stop-signal tasks that test inhibitory control based on button press movements^2^, we chose to explore the feasibility of a gait initiation task which requires a person to quickly initiate gait in response to a go-signal, but to immediately block gait initiation if the go-signal is followed by a stop-signal. This task is analogous to a real-life situation requiring both inhibitory and balance control, with the need to immediately block gait to avoid a collision with an unexpected approaching vehicle.

The neuro-mechanics of gait initiation are well-understood; based on a posterior displacement of the Centre of Pressure (COP) caused by (de)recruiting lower leg muscles^11^, gravity causes a forward momentum of the body’s Centre of Mass (COM) which is followed by a first forward step^12^. The duration of gait initiation depends on the amplitude and timing of posterior COP displacement; a quick and large COP shift causes more forward momentum and a quicker step initiation. Since we aim to evaluate the ability to successfully suppress gait initiation under conditions of different difficulty, we developed a protocol for presenting stop-signals with different delays after a go-signal. We expect that the difficulty of successfully blocking gait depends on the extent to which a person has created a forward momentum by shifting the COP. Thus, we expect that gait inhibition will be more difficult with larger delays of the stop-signal, and that after a certain threshold it will no longer be possible to successfully block gait initiation. With similar delays, we expect young adults (YA) to be more successful than older adults (OA). However, at present it is unclear which latencies are feasible (or not) and whether YA and OA show large differences in task performance. For further studies, it is essential to know the degree to which the task is feasible for YA and OA, as this information is a key criterion for assessment. Therefore, this study aims to evaluate the feasibility of our methodological approach and its relevance in studying age-related changes in motor inhibition during gait initiation. To this purpose, this paper analyses age-related differences in spatio-temporal measures of the COP during all go- and stop-trials, as well as the success of gait inhibition during stop-trials.

## Methods

This study is part of a larger project addressing the impact of IC on the performance of mobility related tasks and was approved by the ethical committee of the German Sport University (ID 095/2021). The methodological approach of the present study was designed based upon initial pilot data from a small number of participants^13^. Methods were performed in accordance with relevant regulations and guidelines.

### Participants

An a-priori sample size estimation using G*Power (version 3.1.9.7) based on step reaction times reported by Magnard et al.^14^ indicated a required sample size of 24 YA and 42 OA for detecting within-group condition effects (with an alpha level of 0.05 and 80% power), and smaller sample sizes for detecting group differences. The final sample of OA was enlarged by also including additional OA who participated in a subsequent pilot project that used the same gait initiation task. After providing written informed consent in accordance with the Declaration of Helsinki, 24 healthy YA, aged between 20 and 35 years (age 26 ± 4 years, 13 female), and 55 OA, aged between 65 and 75 years (age 70 ± 4 years, 26 female) participated in this study. Participants were recruited in Cologne area using an internal advertisement of the German Sport University Cologne as well as leaflet distribution, including a QR code for a brief promotional video. Inclusion criteria were an intact ability to hear and see (with or without assistive devices) and an absence of health conditions that affect mobility and/or balance. Exclusion criteria were acute injuries, chronic diseases, sensory impairments, gait and/or balance deficiencies, and the inability to walk without assistive devices. The exclusion criteria were checked via a questionnaire, which also included other background questions, e.g., with regard to habitual physical activity. In addition, the Montreal-Cognitive-Assessment test was conducted for a cognitive screening; a cut-off score of < 23 was used^15^ as the exclusion criterion.

### Gait initiation-stop task

**Fig. 1:**
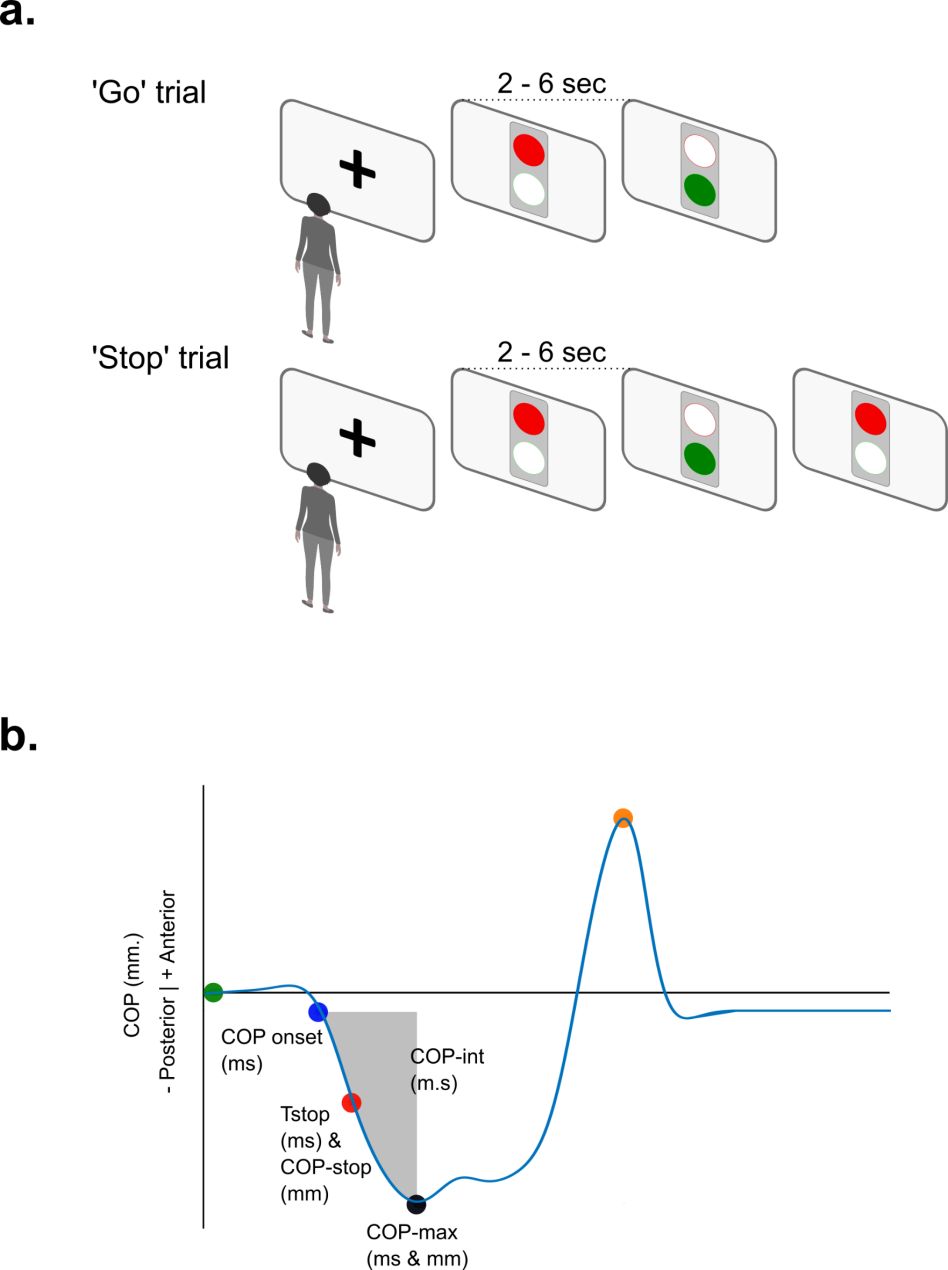
Key events during the gait initiation-stop task. (**a**): Schematic illustration of the sequence of on-screen signals during go- and stop-trials of the gait initiation stop task. (**b**): typical changes in antero-posterior COP position during gait initiation. After a go-signal (green dot), gait is initiated by a posterior shift of the COP (blue dot). Maximum posterior displacement (COP-max (black dot)), is followed by a toe-off of the leading leg (orange dot). In addition to these events, the figure indicates the COP integral until COP-max (grey area), and an example of the timing of a stop-signal (red dot) during stop-trials.

While standing on a force plate, a person was instructed to stand quietly and focus on a black ( +) signal presented on a large screen (0.52 by 0.93 m) approximately 4 m in front of the person. After the ( +) changed into a virtual traffic light, the person was required to quickly initiate gait and walk 4 m when the light turned to green (see ‘Go’ trial in Fig. 1a). In some of the trials, the green signal unexpectedly changed to red (see ‘Stop’ trial in Fig. 1a), which prompted the person to immediately block gait initiation and maintain the initial upright standing position on the force plate while not making a step.

### Instruments and data preparation

Ground reaction forces and kinematic data of anatomical landmarks were obtained by a Bertec force plate (1000 Hz) and a 3D motion analysis system (Qualisys AB, SE) operating at 100 Hz. The green go- and red stop-signals were triggered based on custom-made Matlab scripts based on Psychtoolbox-3 (MATLAB, R2022a, MathWorks, Natick, MA). The ground reaction forces and the COP signal were filtered using a second-order, recursive Butterworth filter with a cut-off frequency of 5 Hz.

### Measurement protocol

Before the start of an experiment, a participant completed 5 trials to determine the habitual gait initiation response to a go-signal. From these initial trials, the average duration from the go-signal to the first posterior peak of the COP changes preceding step initiation was determined. This individual average peak timing was used to define 3 different latencies for presenting a red stop-signal after a green go-signal (i.e. a long-latency, which was equal to the average peak timing; a medium-latency, equal to peak timing minus 150 ms; and a short-latency, equal to peak timing minus 300 ms). The magnitude of these 3 latencies was chosen based on the initial pilot-data^13^.

After determining the 3 individual latencies, a person was asked to perform 3 blocks of 12 gait initiation trials. Each block comprised a randomized sequence of 9 go- and 3 stop-trials comprising each of the 3 latencies. Thus, after completing the three blocks, 3 stop-trials were available for each latency. For all trials, the ‘wait time’ before presenting a green go-signal was randomized between 2 and 6 s (see Fig. 1a). Between blocks, participants had a short break (ca. 3–5 min). During these breaks, participants were allowed to sit down and rest, and, if needed, take extra time.

### Data analyses

Figure 1b indicates key events for evaluating the performance of the gait initiation stop task. The following variables were determined for further analysis:

- Timing of go- (**Tgo**) and stop-signals (**Tstop**) as generated by the Matlab script using the Psychtoolbox-3;
- Gait initiation as defined by the onset of COP displacement (**COP-onset** was the instant after a go-signal where a posterior shift in COP exceeded 5 mm);
- **Stop-latency**, the time difference between gait initiation and a stop-signal (**Stop-lat** equals Tstop– COP-onset);
- For all trials, the amplitude and timing of maximum posterior COP displacement (**COP-max**), as well as an estimate of the COP integral at COP-max **(COP-int)**;
- For all stop-trials, the amplitude **(COP-stop)** and integral of COP displacement **(COP-stop-int)** at the instant of presenting a stop-signal (i.e., at Tstop); in addition, a relative COP integral was calculated that expressed COP-int during a stop-trial relative to the mean COP-int during all go-trials (i.e., **StopGo-rel-int** equals *100 x (COP-int of a stop-trial – mean COP-int of all go-trials) / (mean COP-int of all go-trials)*;
- Finally, any forward movement of the left or right toe for more than 30 mm was used as a quantitative criterion for **success or failure** in inhibiting gait after receiving a stop signal.

### Statistical analysis

A first descriptive data-analysis comprised means and standard deviations (SD’s) of spatio-temporal COP variables during the 5 initial gait trials, and during all go- & stop-trials. Using data from all stop-trials, survival curves were created to indicate success of gait inhibition (calculated as the percentage of successful stop trials) in dependency of Stop-lat, COP-stop and COP-stop-int. Subsequently, receiver operating characteristic (ROC) curves and their corresponding area under the curve (AUC) were examined to determine the discriminative power of Stop-lat, COP-stop, and COP-stop-int. Euclidian distance was used to determine cut-off values that separated successful and unsuccessful stop trials with optimal sensitivity (sens) and specificity (spec)^16^. The final statistical analysis then focused on selected data of YA and OA under conditions of similar difficulty. Binary logistic regression models including Stop-lat, COP-stop, COP-stop-int as well as relative COP integral were used to analyze (un)successful performance. Age-related differences between these variables were assessed using the Mann–Whitney U test.

## Results

All YA and OA were able to complete the initial gait initiation trials as well as the subsequent blocks of go- and stop-trials. However, some individual trials were not in accordance with instructions. Trials were excluded if: gait was initiated before a go-signal; if a response was unlikely fast (i.e. COP-onset <  = 125 ms); if gait initiation was extremely late (COP-onset >  = 550 ms); or if there was no response at all. Ultimately, 617 out of 711 stop-trials (87%) were completed according to instructions; 16 trials were excluded in YA (7%), and 78 trials in OA (16%).

### COP displacement during initial gait initiation trials and go-trials

During the initial 5 gait initiation trials, the mean duration to COP-onset and the time to maximum posterior COP displacement were similar in YA and OA, however the amplitude of posterior COP displacement as well as the COP integral were somewhat lower in OA (see Table 1 left panel). A comparison of go-trials in the three successive blocks of mixed go- and stop-trials did not show a block effect on the timing of gait initiation (repeated measures ANOVA for COP-onset: *p* = 0.94). Hence all available go-trials (i.e. maximally 3*9 individual trials) were used for calculating individual means and then group mean data. During the go-trials, participants tended to initiate gait later and with a smaller and slower posterior shift than during their initial gait trials (see Table 1 left panel). These changes in COP shifts between initial- and go-trials were somewhat more pronounced in YA.

**Table 1:** Spatio-temporal measures of COP displacement during all go- and stop-trials.

	Initial trials	Go-trials		Stop-trials		
				All	Success	Failure
COP onset (ms)	**246.3** (27.9)	**310.8** (45.4)	**YA**	**310.1** (79.8)	**355.7** (91.2)	**291.9** (66.9)
	**259.4** (48.2)	**302.1** (62.8)	**OA**	**287.5** (80.4)	**339.4** (105.6)	**275.3** (68.0)
Tstop (ms)	n.a	n.a	**YA**	**494.8** (146.5)	**372.6** (108.5)	**543.5** (130.7)
	n.a	n.a	**OA**	**477.0** (144.0)	**355.4** (111.4)	**505.5** (135.8)
Stop-lat (ms)	n.a	n.a	**YA**	**184.7** (174.8)	**16.9** (144.3)	**251.6** (137.1)
	n.a	n.a	**OA**	**189.5** (172.4)	**15.9** (138.2)	**230.1** (153.4)
COP-stop (mm)	n.a	n.a	**YA**	**-41.7** (34.7)	**-5.6** (14.9)	**-56.2** (29.5)
	n.a	n.a	**OA**	**-42.7** (33.5)	**-6.4** (14.5)	**-51.1** (31.0)
COP-stop-int (m.s)	n.a	n.a	**YA**	**-11.8** (12.4)	**-1.6** (3.1)	**-15.9** (12.3)
	n.a	n.a	**OA**	**-12.3** (12.4)	**-1.4** (2.4)	**-14.8** (12.4)
COP-max (ms)	**638.1** (100.3)	**775.6** (123.3)	**YA**	**656.5** (126.4)	**596.3** (105.3)	**680.4** (126.4)
	**640.5** (93.2)	**728.4** (107.2)	**OA**	**633.8** (126.0)	**592.7** (109.5)	**643.4** (127.8)
COP-max (mm)	**97.8** (18.3)	**85.5** (19.9)	**YA**	**-74.7** (28.9)	**-50.7** (21.8)	**-84.3** (25.6)
	**88.6** (15.7)	**84.1** (15.9)	**OA**	**-77.9** (22.9)	**-51.2** (20.8)	**-84.2** (18.5)
COP-int (m.s)	**38.0** (11.1)	**39.5** (11.4)	**YA**	**-27.4** (16.1)	**-13.6** (10.1)	**-32.9** (14.8)
	**33.8** (10.0)	**36.2** (11.0)	**OA**	**-28.0** (15.0)	**-13.3** (7.4)	**-31.5** (14.2)

Spatio-temporal measures of the Centre of Pressure (COP) displacement after go-signals during initial gait initiation trials (n = 5), as well as during all go- (n = 3*9) and stop-trials (n = 3*3). Data of stop-trials also indicate timing of the stop-signal (Tstop) after a go-signal, as well as COP-amplitude and -integral at Tstop. Data comprises means and standard deviations of young (YA (n = 24)) and old adults (OA (n = 55)).

Mean values are presented in bold, with standard deviations in parentheses.

### Inhibition of gait initiation during stop-trials

Given that stop-signals were presented with 3 pre-determined fixed delays after go-signals and subjects were variable in the timing of their responses to a go-signal, stop-signals arrived with a variable timing during gait initiation. As most subjects responded slower to a go-signal during the three blocks of trials, stop-signals often arrived early during gait initiation, and could in fact, even arrive -before- the start of COP displacement (i.e., before COP-onset).

Table 1 (right panel) shows group mean data of all stop-trials, as well as of (un)successful trials. Over all stop-trials, the stop-signal conditions did not significantly differ between YA and OA, but YA were able to successfully inhibit gait initiation in 57 out of 200 trials (29%), whereas OA were successful in 79 out of 417 stop-trials (19%). The data in Table 1 show consistent differences between successful and unsuccessful stop-trials in YA as well as OA. On average, the start of gait initiation (COP onset) was later and the arrival of stop-signals (Tstop) earlier, and most importantly, the COP displacement and COP integral at the instant of a stop-signal were considerably smaller during successful stop-trials. These data highlight that in both age groups successful inhibition was primarily limited to conditions with minimal instability, i.e., trials in which a stop-signal arrived early during gait initiation. Relative to gait initiation, the latency of stop-signals (Stop-lat) showed positive and negative values. Over all 617 stop-trials, Stop-lat varied between -308 to + 622 ms. The mean latency of the stop-signals did not substantially differ between YA and OA, and neither did the amplitude and integral of COP displacement at Tstop (see mean data of all stop-trials in Table 1 (right panel)).

**Fig. 2:**
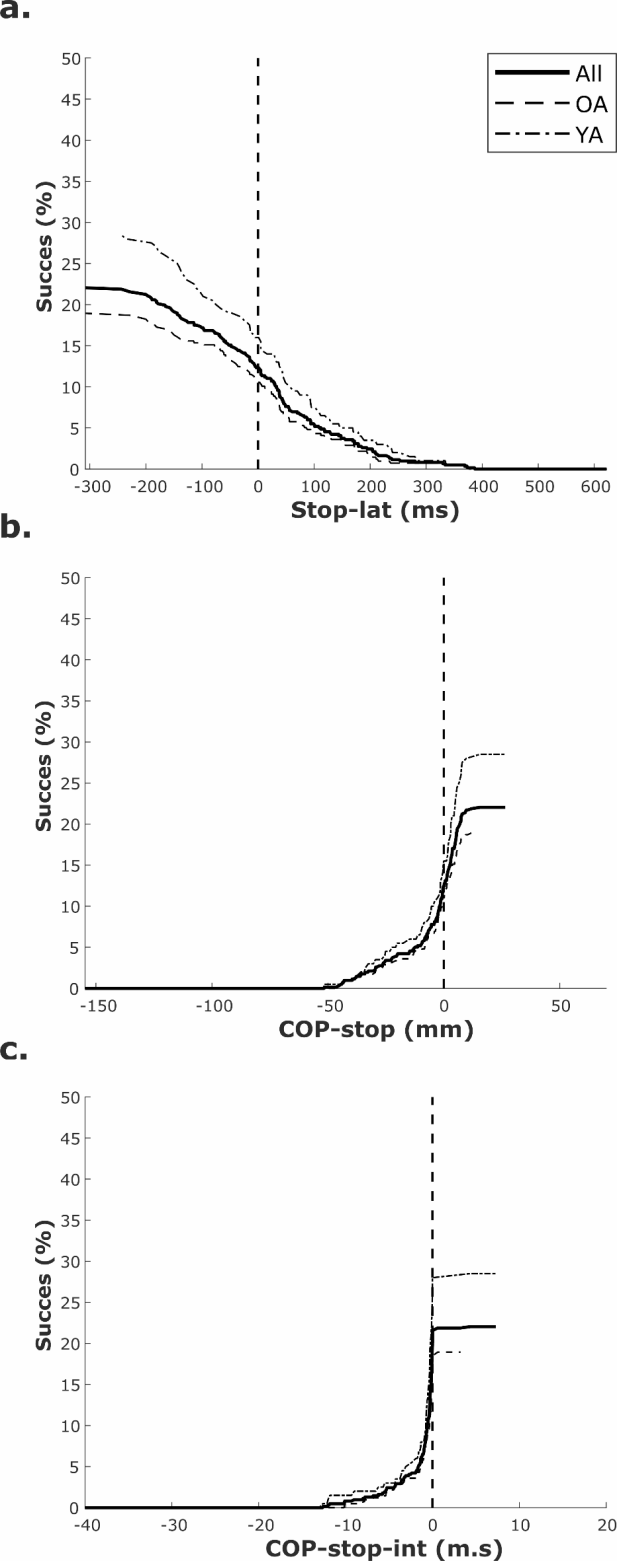
Success rate during stop-trials in young and old adults. Percentage of successful stop-trials (Success rate) in dependence of: timing of the stop-signal relative to onset of COP displacement (Stop-lat; see **a**.) as well as posterior COP displacement (COP-stop; see **b**.) and COP integral (COP-stop-int; see c.) at the instant a stop-signal was presented. Solid lines indicate data of all participants, and dashed lines indicate data of young (YA) and old adults (OA). In all figures, the vertical dashed lines indicate values at COP onset.

Figure 2 shows survival plots that indicate the proportion of remaining successful trials (success rate) in dependency of latency of the stop-signal (Stop-lat (top panel)), magnitude of COP displacement (COP-stop (middle panel)) and COP-stop integral (lower panel) at the time of presenting a stop signal. In both age groups, success rate decreases with increases in Stop-lat, and decreases in COP-stop, or COP-stop integral. The figures indicate that the ability to successfully inhibit gait initiation, i.e. not make a forward step, remains large when stop-signals were presented before, or shortly after gait initiation, when COP displacement and COP integral still were small. However, the success rate steeply declines with further increases in latency, COP displacement and COP integral, and beyond certain thresholds there are no remaining successful trials. Decreases in success rate showed similar trends in YA and OA, but the figures indicate that the success rate was consistently lower in OA.

### ROC-analysis of stop-trials

**Fig. 3:**
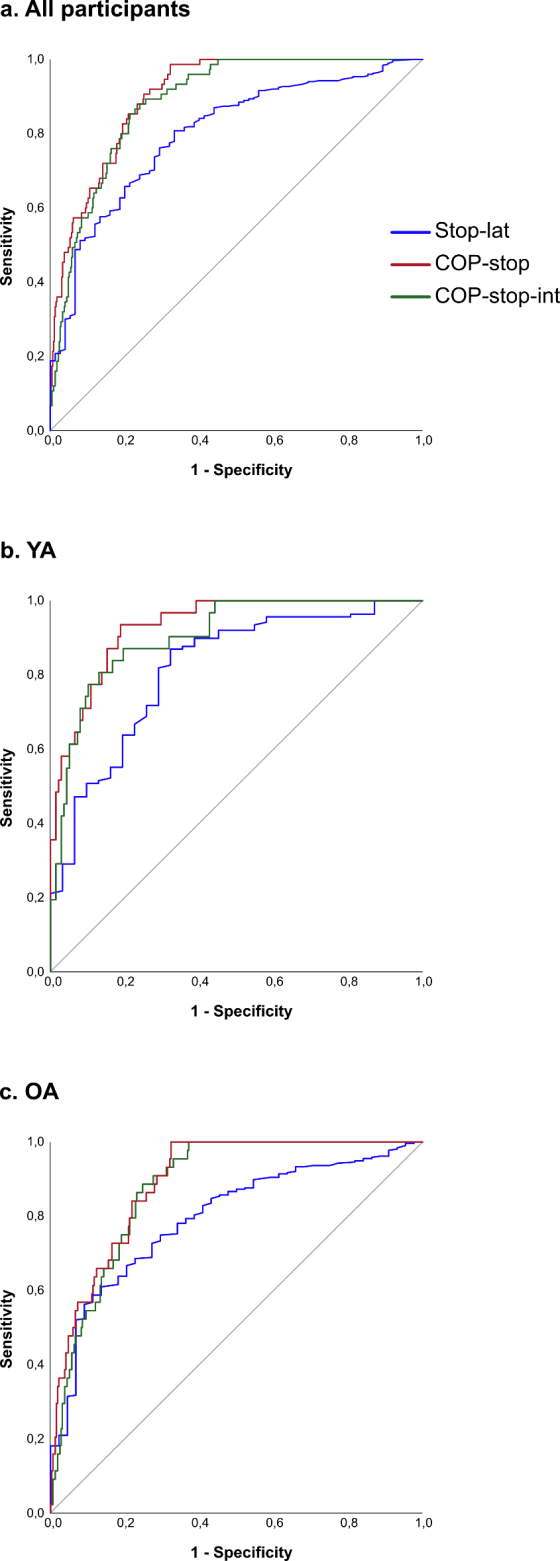
ROC curves discriminating successful from unsuccessful stop-trials. ROC curves illustrating the discriminating power of Stop-latency (blue), COP-stop (red), and COP-stop-int (green) for distinguishing between successful and unsuccessful stop-trials. A curve that closely follows the left-hand border and top border of the ROC space indicates higher accuracy. Panel a ROC curves for data of all participants, and panel b and c are based based on data in young (YA) and older adults (OA) respectively.

In individual data of all stop-trials, it was observed that when stop-signals arrived before COP onset (i.e., with negative latencies) the majority of trials were successful in YA as well as in OA (though again with more success in YA (see data in Fig. 2)). However, the individual data also showed that neither young nor old persons were able to be successful when stop-signals were presented beyond certain thresholds (see the changes in success rate with Stop-lat, COP-stop, and COP-stop-int as presented in Fig. 2). To determine cut-off values for optimally separating successful from unsuccessful stop-trials, ROC analyses focused on Stop-lat, COP-stop, and COP-stop integral using all available stop-trials in which a stop-signal arrived after the onset of gait initiation (i.e., positive latencies only). These data comprised 44 successful and 315 unsuccessful stop-trials in OA (i.e., a success rate of 12.2%), and 31 versus 138 in YA (18.3%). COP-stop showed the highest AUC of 0.90, followed by COP-stop integral with 0.89, and Stop-lat with 0.80, based on all trials from both age groups (see Fig. 3). Respective cut-off values were **-34 mm** for COP-stop (sens/spec = 0.85/0.79), **-5.6 m.s** for COP-stop integral (sens/spec = 0.85/0.79), and **149 ms** for Stop-lat (sens/spec = 0.76/0.71). Age-specific values for COP-stop, were -38 mm in YA (sens/spec = 0.94/0.81, AUC = 0.93) versus -30 mm in OA (sens/spec = 0.84/0.78, AUC = 0.89). For COP-stop integral, these values were -5.6 m.s in YA (sens/spec = 0.84/0.83, AUC = 0.90) versus -5.6 m.s in OA (sens/spec = 0.84/0.83, AUC = 0.88), and for latency the cut-off values were 145 ms in YA (sens/spec = 0.82/0.71, AUC = 0.82) versus 155 ms in OA (sens/spec = 0.73/0.73, AUC = 0.80).

### Age-related differences in inhibitory performance under conditions of similar difficulty

The previous sections reported consistent age-related differences in performance. However, the comparison of the inhibitory performance of old versus young subjects comprised the full range of individual responses to stop-signals. The timing of individual responses resulted in different degrees of difficulty of presented stop-signals, and may bias the comparison between age-groups. Therefore, additional analyses of the inhibitory performance of OA versus YA focused on conditions of similar difficulty based on selected trials (i.e., only stop-trials with positive latencies and COP-stop > -34 mm). Binomial logistic regression models to predict successful inhibition in both age groups (see Table 2) were statistically significant (YA: χ^2^(4) = 24.1, *p* < 0.001; OA: χ^2^(4) = 18.9, *p* < 0.001), and indicated a very large effect size for the model in YA (*f*^2^ = 1.13) and a medium-to-large effect size for the model in OA (*f*^2^ = 0.28). Overall, 85.4% of trials were correctly classified by the YA model and 76.6% by the OA model. Only relative integrals of COP in stop- compared to go-trials contributed significantly to predicting success with the effect being more pronounced in YA than in OA (see Table 2).

**Table 2:** Logistic regression models to predict successful inhibition.

Group	Predictor	*B*	*SE*	t-Statistic	*p*
YA	Intercept	-1.59	1.40	-1.14	.25
	Stop-lat (ms)	-0.00	0.01	-0.02	.98
	COP-stop (mm)	0.08	0.06	1.29	.20
	COP-stop-int (m.s)	-0.10	0.37	-0.28	.78
	StopGo-rel-int	-0.06	0.02	-2.99	*.00*
OA	Intercept	-0.93	0.62	-1.50	.13
	Stop-lat (ms)	-0.00	0.00	-0.28	.78
	COP-stop (mm)	0.07	0.04	1.67	.09
	COP-stop-int (m.s)	-0.09	0.20	-0.42	.68
	StopGo-rel-int	-0.03	0.01	-2.83	*.00*

Results of logistic regression predicting binary motor inhibition outcomes (success vs. failure) using four predictors: timing of the stop-signal (Tstop) after a go-signal (Stop-lat), COP-amplitude and -integral at Tstop (COP-stop, COP-stop-int), as well as relative integral (StopGo-rel-int) in young adults (YA) and older adults (OA). These analyses were based on a total of 159 trials included, with 48 from YA and 111 from OA. The success rate was 56% in YA and 33% in OA.

Additional statistical analyses showed significant differences between age groups in success rate (*U* = 2053, *Z* = -2.7, *p* < 0.05) and StopGo-rel-int (*U* = 2116, *Z* = -2.1, *p* < 0.05), but no significant differences for Stop-lat (*U* = 2606, *Z* = -0.2, *p* = 0.83), COP-stop (*U* = 2622, *Z* = -0.2, *p* = 0.88), or COP-stop-int (*U* = 2646, *Z* = -0.1, *p* = 0.95).

## Discussion

This study analyzed the results of a novel stop-signal task to study inhibitory control during gait initiation in YA and OA. Our assessment protocol included conditions of different difficulty, and our overall expectation was that, given similar difficulty, YA would be more successful than OA. Given the novelty of our task and the absence of similar studies, our primary aim was to evaluate the feasibility of our methodological approach and its relevance for studying age-related differences in motor inhibition during gait initiation. This discussion section will first address the feasibility of our approach, then analyses task-performance and age-related differences during go- as well as stop-trials, and then address the relevance of the presented approach for studying inhibitory control and providing an outlook on further studies of motor inhibition.

### Feasibility of the methodological approach

The many repeated trials (i.e., 41 gait initiation trials per person) that were part of the assessment protocol proved to be feasible in YA as well as in OA; all participants were able to complete the protocol without any difficulties. Compared to common Stop-signal tasks^17^, our protocol was similar in terms of the proportion of stop-trials (i.e. 3 on 12 (25%) per block), but had a small number of stop-trials (3*3 in total). A lower ratio (e.g. ≤ 20%) may increase randomness and potentially lower the anticipation of stop-trials^18,19^. However, lower ratios require a substantial increase in total number of trials to arrive at the same number of stop-trials. Whereas a large number of trials is usual and easy to achieve in common Stop-signal tasks where subjects remain seated, it is likely to induce undesired effects such as fatigue or a reduced attention during repeated gait initiation trials, which may influence performance^20^. In our study, comparing the three blocks of trials did not show significant changes and our participants did not need long breaks between blocks. Thus, it seems fair to conclude that our protocol did not induce fatigue in our participants. Nevertheless, the total number of gait trials is an important issue to be considered in further studies, especially when analyzing performance in less fit OA or in patients with pathology that affects balance and mobility. An additional feasibility aspect of our methodological approach is task difficulty and inhibitory success. We aimed to have three difficulty levels by providing stop-signals at three different latencies and we defined success as the ability to completely block a step (i.e., no forward movement of either foot). Our results show low overall success rates in YA as well as OA, and neither old nor young persons were successful beyond certain stop-signal conditions. Hence, it is essential to limit an experimental protocol to stop-signals which are at least potentially feasible for old and young subjects. Considering the abovementioned feasibility aspects, it seems important that studies of gait inhibition rather focus on one well-chosen condition with identical and feasible stop-signals than on multiple conditions. Experiments focusing on one condition could potentially have a lower ratio of stop-trials as well as more repetitions (e.g., 3 blocks of 15 trials at a 20% ratio can yield 9 repetitions of identical stop-signal conditions), and thus provide a reliable assessment. After addressing our results in detail in the following sections, a final section will provide concrete suggestions for how feasibility aspects can be taken into account in further studies.

### Adaptive behavior during go- and stop-trials

Our results (see Table 1) show that during initial gait trials the timing of gait initiation was almost identical in YA and OA (i.e., COP onset after ca. 250 ms and maximum posterior displacement at ca. 640 ms), whereas the amplitude and integral of COP displacement were slightly lower in OA (COP-max: 98 mm in YA and 89 mm in OA; COP-int: 38 m.s in YA and 34 m.s in OA). Since there was no significant block-effect, individual means of COP variables were calculated over all available go-trials. The group mean data of all go-trials shows similar age-differences as the initial gait trials. However, a notable finding in the go-trials of YA and OA is a consistently later COP-onset, as well as a later and smaller posterior COP displacement than during the initial gait trials (see Table 1). We interpret these consistent changes as pro-active adaptive behavior which made it easier to be successful in stop-trials: first, with a later response to a go-signal, a stop-signal is more likely to arrive before or shortly after the onset of COP changes (i.e., with a more advantageous timing); second, with a more cautious COP displacement, the change in forward momentum and ensuing instability are less drastic^21^. Thus, these two aspects can be interpreted as elements of a pro-active strategy to make it easier to successfully block gait and remain standing. This pro-active strategy was observed in YA as well as in OA, but was more pronounced in YA and may have contributed to their larger success in inhibiting gait.

### Success of gait inhibition during stop-trials

Our expectancy that the difficulty to successfully block gait initiation depends on the extent to which a person has created a forward momentum by a posterior displacement of the COP is confirmed by our results: the consistent differences in mean COP variables between successful and unsuccessful stop-trials (Table 1) highlight that successful trials are characterized by minor COP displacement and COP integral when the stop-signal arrived, and that in unsuccessful trials these variables were larger; Fig. 2 highlights the dependency of success rate from stop-signal latency, COP displacement and COP integral respectively; and the presented ROC analyses yielded cut-off values that indicate that especially the amplitude of COP at Tstop (i.e., -34 mm as determined from data of both age groups) dissociates success from failure. Though our overall findings confirm that gait inhibition is more difficult with larger delays of the stop-signal, it must be noted that stop-signal latency by itself is not the essential critical factor; with increasing latency, posterior displacement and COP integral are larger and these latter variables cause a forward momentum and instability which at some point cannot be reversed. Persons can be slow or quick in displacing COP. Hence, instability is directly related to COP displacement and COP integral, but only indirectly related to stop-signal latency.

Overall, we observed similar trends in YA and OA: i.e., except for maximum COP displacement, initial gait initiation trials were very similar; and, during the blocks of go- and stop-trials, both YA as well as OA showed the same tendency to have a delayed gait initiation and slower and smaller COP displacements compared to initial trials. Despite such adaptive behavior, the success rate remained low in YA (29%) as well as OA (19%), but overall, YA were consistently more successful than OA. Nevertheless, it should be noted that an accurate analysis of age-related differences in motor inhibition during gait initiation should focus on motor inhibition in conditions of similar difficulty. To this purpose, we used the cut-off value suggested by our ROC analysis. Our analyses of selected stop-signal trials demonstrate that in conditions of similar difficulty only relative COP integral significantly predicted success and that OA are significantly less effective in motor inhibition than YA. The analyses emphasize that the ability to inhibit further COP displacement after a stop-signal is essential for successful inhibition of gait initiation, and that the reduction of the COP integral relative to go-trials can be considered indicative of motor inhibition. Thus, when considering findings in all go- and stop-trials of our gait inhibition task, our results highlight that inhibitory success was associated with two strategies: a general pro-active strategy involving cautious COP displacement; and a motor inhibition strategy that consisted of an inhibition of further COP displacement after a stop-signal.

### Relevance of our methodical approach and an outlook on further studies of motor inhibition

Our methodological approach proved to be feasible and our a-priori expectations regarding task difficulty and age-effects were confirmed by our results. However, our findings stress the importance of using a protocol that presents stop-signals within a range that may be challenging, but where success is feasible. Presenting stop-signals with fixed latencies after a go-signal leads to a highly variable timing of stop-signals in relation to the start of gait initiation due to a variable timing of COP displacements and adaptive strategies to reduce task difficulty. To solve this issue, stop-signals can be triggered based on the real-time behavior of participants; e.g., with real-time monitoring of the COP, a stop-signal can be presented when COP displacement crosses a certain threshold. Thus, it would be possible to present stop-signals with an exact timing after the start of gait initiation, or with selected COP amplitudes. Especially the latter solution seems to offer a fair option for comparing subjects’ behavior under conditions of identical (in)stability, and it further allows for evaluating subjective reports about task difficulty. Our results suggest -34 mm as a lower limit for triggering stop-signals. However, it should be kept in mind that our in-exclusion criteria targeted healthy and fit YA and OA; in other groups of OA, e.g., with mobility limitation and/or an increased fall risk, there may be the need to choose less challenging conditions.

For further studies, it is essential to consider the neuro-mechanical sequence of events associated with gait initiation and/or motor inhibition. Gait initiation, particularly under constant attentional control, is associated with preparatory brain activity^22,23^ followed by specific muscle recruitment patterns, which lead to mechanical changes (i.e., a COP shift and subsequent initiation of a first step^11,12,24^). Studies show that before COP onset the activity of m. Soleus is inhibited, and this inhibition is followed by bilateral excitation of m. Tibialis Anterior^11^ but the extent to which this activation pattern is present varies strongly between persons and is affected by initial posture^25^. The muscle activity leads to a COP displacement and increasing forward momentum and instability^24^, and inhibitory control processes must act on muscle recruitment and reverse the mechanical changes before a point-of no-return where instability is too large to be corrected. Thus, in our gait initiation task, inhibitory control can be quantified based on variables that indicate changes in brain activity, muscle activity, COP position, mechanical (in)stability, and ultimately the success of blocking gait initiation. Our study focused on spatio-temporal measures of COP displacement and success rate as indicated by maintaining foot position. Based on our results, we suggest that the contrasts in COPmax-amplitude and -integral between stop- and go-trials indicate motor inhibition. Further studies of inhibitory control during gait initiation may specifically focus on an analysis of brain or muscle activity and/or mechanical stability (e.g. as indicated by Margin of Stability^26,27^). Given the scarcity of similar studies, it is important that further studies also include different conditions of IC during balance tasks, e.g., involving perceptual and motor inhibition^7,28^, and relate their results to measures of physical and cognitive functioning. Ultimately, we expect such studies help us better understand the relationships between changes in cognition and balance performance, and their impact on daily life mobility^29,30^ and fall risk^8,31,32^.

### In conclusion

The results of our study show the feasibility of a new stop-signal gait initiation task for assessing motor inhibition in YA and OA. Our analyses highlight two adaptive strategies that were observed in YA as well as OA: a pro-active strategy, which involved a delayed and slower shift of COP movements in go- and stop-trials compared to initial trials; and motor inhibition, which involved the inhibition of COP shifts after a stop-signal. Our results indicate that beyond a certain threshold of COP displacement, successful task performance was neither feasible in YA nor OA. Overall, our results show age-related differences that indicate typical changes in gait initiation in OA as well as a reduced ability to successfully block gait initiation in response to stop signals. Our findings highlight an age-related decline in motor inhibition and provide essential information for the development of novel assessment tools as well as well-focused studies that aim to further investigate neuro-mechanical aspects of motor inhibition.

## Data Availability

Data availability statement Data in this manuscript are not publicly available, but access to the data is possible upon request to the corresponding author.
